# Development and validation of a tool to measure patient experience in chronic disease care

**DOI:** 10.4102/phcfm.v10i1.1830

**Published:** 2018-09-11

**Authors:** Nayna Manga, Richard Harding, Angela de Sa, Kathleen Murie, Mosedi K. Namane, Peter J. Raubenheimer, Derek A. Hellenberg, Elma de Vries

**Affiliations:** 1 Division of Family Medicine, School of Public Health and Family Medicine, University of Cape Town, South Africa; 2 Centre for Global Health Palliative Care, King’s College London, United Kingdom; 3 Retreat Community Health Centre, Cape Town, South Africa; 4 Cape Town Metro District Health Services, Western Cape Department of Health, South Africa; 5 Vanguard Community Health Centre, Cape Town, South Africa; 6 Division of General Internal Medicine, University of Cape Town, South Africa; 7 Heideveld Community Health Centre, Metro District Health Services, South Africa

## Abstract

**Background:**

There is a global increase in the prevalence of non-communicable diseases and a growing understanding that patients need to be involved in their care. Patient experience should be assessed and the information used to improve on the planning and delivery of health services.

**Aim:**

This study described the development and validation of a patient-reported experience measure (PREM) tool which is appropriate for the South African context, to assess self-reported patient experience of chronic care.

**Setting:**

The study was conducted at four primary health care facilities in the Cape Town Metropole.

**Methods:**

This was a validity and reliability study with multiple phases to develop and determine the psychometric properties of a novel tool. It consisted of three phases, namely: Phase 1 – Consensus Validity; Phase 2 – Face Validity; Phase 3 – Reliability. Phase 1 consisted of an expert panel reaching consensus on a draft tool. Phase 2a consisted of qualitative semi-structured interviews and cognitive interviews. Phase 3 tested the internal consistency of the tool, the time necessary to complete, as well as floor and ceiling effects with 200 questionnaires.

**Results:**

The process described resulted in a final questionnaire with *n* = 10 items in three languages that was easily understood by patients. Internal consistency was determined with the overall Cronbach’s alpha 0.86. This PREM has been named Chronic Care Assessment of Patient Experience.

**Conclusion:**

Using best practice guidance in tool construction and validation, we delivered a PREM with the potential to improve the quality of care from the perspective of patients. Implementation studies are now required to determine how best to use this tool in routine practice.

## Introduction

The global increase in the prevalence of non-communicable diseases (NCDs) has disproportionally affected low- and middle-income countries such as South Africa, with 80% of NCD deaths worldwide occurring in these countries.^1^ The burden of disease related to NCDs is predicted to increase substantially in South Africa over the next few decades if measures are not taken to combat the trend.^2,3^ Furthermore, the health and socio-economic toll of the NCD epidemic is impeding achievement of the millennium development goals, which are falling short of targets set in many countries. NCDs are also increasingly contributing to premature deaths;^4^ in South Africa, NCDs formed 60% of the 10 leading underlying natural causes of death, requiring an integrated model of quality care to retain people with chronic disease from diagnosis until the end of life.^5^ In addition to diabetes mellitus, health problems such as cerebrovascular diseases, other forms of heart disease, hypertensive diseases, chronic lower respiratory diseases and ischaemic heart diseases contributed to the rise in NCDs.^6^ In the Metropole of the Western Cape province of South Africa, NCDs were found to account for 82% of visits to Community Health Centres (CHCs).^7^

In 2011 there was extensive global focus on NCDs culminating in the United Nations General Assembly High Level Meeting of Heads of State and Governments and the adoption of the Political Declaration on the Prevention and Control of NCDs. The South African National Department of Health published a strategic plan for the prevention and control of NCDs in 2013 which committed to achieving 10 goals to be achieved.^8^ One of the goals is to ‘increase the percentage of people controlled for hypertension, diabetes and asthma by 30% by 2020’.

Unfortunately, the majority of physicians and patients report that it is difficult to obtain high-quality care for chronic illnesses.^9^ An integrated audit of chronic NCD management has been performed annually in the Western Cape from 2009. The audit tool measures structural, process and intermediate outcome indicators for diabetes, hypertension, asthma, chronic obstructive pulmonary disease (COPD) and epilepsy. Improvements have been shown in chronic care processes.^10,11^ Whilst some gains in clinical quality for chronic disease care have been achieved, there is still substantial room for improvement.

Although several approaches have been utilised to translate evidence-based recommendations into clinical practice, the chronic care model (CCM) has been the most effective model that has been implemented in a variety of health care settings internationally, often with diabetes as the focus disease.^12^ The CCM proposes that the productive interactions of a prepared proactive practice team and an informed, empowered patient and family will lead to improved outcomes. An activated patient is one who has the motivation, information, skills and confidence necessary to make self-management decisions about their diabetes. Patients bring unique and important perspectives on their own care, on the experience in health care organisations and on the coordination and cooperation amongst various elements of their care. Unfortunately, patients, their families and other caregivers, as well as the public all too often are not meaningfully engaged in care or as partners in its improvement. Moving to the vision of a system centered on people’s needs and preferences has the potential to bring multiple benefits to patients, the health care system and the nation. As a result, patient experience and⁄or satisfaction should be assessed and the information used to improve on the planning and delivery of health services.^13,14^ It has been shown that feedback to health care providers regarding the specific care or results of care received or experienced by their patients can result in significant improvements.^15^ The strategic direction of the Western Cape Department of Health, expressed in *Healthcare 2030 – the road to wellness* recognises the importance of person-centred care.^16^

We aimed to develop and validate a patient-reported experience measure (PREM)^17^ to assess self-reported patient experience of chronic care as a person-centred experience in South Africa. There is increasing international attention regarding the use of PREM as a quality indicator of patient care and safety. This reflects the ongoing health service commitment of involving patients and the public within the wider context of the development and evaluation of health care service delivery and quality improvement.^17^

## Methods

**FIGURE 1:**
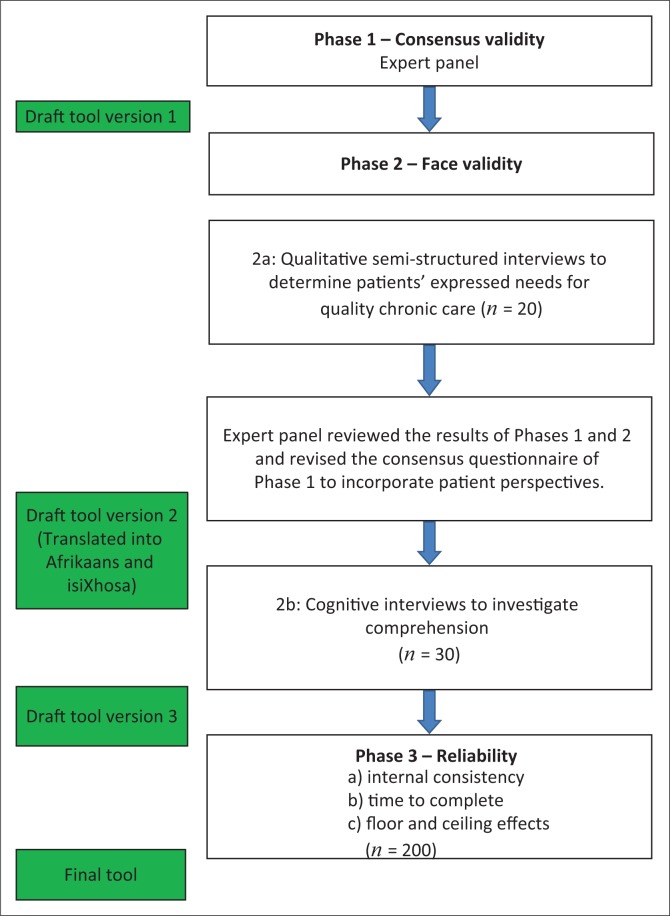
Phases of the study.

This is a validity and reliability study with multiple phases to develop and determine the psychometric properties of a novel tool. It consisted of three phases, namely: Phase 1 – Consensus Validity, Phase 2 – Face Validity, Phase 3 – Reliability (Figure 1).

### Phase 1: Consensus validity

A focus group consisting, of an expert panel of an internist, six family physicians and a researcher, held a meeting on 13 August 2012. The seven experts were clinicians working in primary, district and tertiary public health facilities in the Western Cape province. The participants had evaluated the items in the Primary Care Assessment Tool (PCAT)^18,19^ and Patient Assessment of Chronic Illness Care (PACIC)^20,21,22^ tool individually before the meeting. In the discussion, the items were considered and the panel debated the applicability of the questions to the local South African context. Issues applicable to chronic care within the tools, such as continuity of care and communication, were considered, whilst issues that were not common practice in South Africa were not considered as suitable questions. An example of a question that would not be suitable would be, ‘Contacted after a visit to see how things were going’. PCAT^18,19^ consists of 100 questions and PACIC^20,21,22^ consists of 20 questions. The aim was to develop a short tool that was easy to administer. Consensus was reached on 11 questions to form the draft tool version 1, with all panel members agreeing. It was decided that a pictorial Likert response scale with ‘smiley faces’ would be the most appropriate format. The scale has five options, namely very satisfied, satisfied, neutral, dissatisfied and very dissatisfied. This is an easy way for patients, who may not be highly literate, to express their views. This type of scale has been successfully used as a tool to assess patient satisfaction at the primary care level in Scotland.^23^

### Phase 2: Face validity

The objective of Phase 2 was to determine whether patients with NCDs felt the tool reflected their views on aspects of patient experience that matter, interpretability and acceptability of the measure to the target population.

#### Sampling

The study’s patient population consisted of patients with chronic diseases (diabetes, hypertension, epilepsy, asthma or COPD) who received care at primary care facilities in the Cape Town Metropole and were 18 years or older. The interviews were carried out in four CHCs in the Cape Town Metropole in Khayelitsha site B, Retreat, Vanguard and Guguletu between September 2013 and August 2014. Inclusion criteria were patients who regularly attended the CHC for chronic disease care and whom the interviewer deemed would be able to give a rich account of their experience. Exclusion criteria were documented as significant cognitive impairment that prevented informed consent. Purposive sampling was used by the interviewers on the day of the interview to select patients with the following characteristics: age, gender, diagnosis. The patients selected had not been consulted by the interviewer prior to the interview.

#### Data collection

Twenty qualitative semi-structured interviews were conducted by the resident family physicians at the four CHCs (five interviews per CHC) to describe patient-expressed needs for quality chronic care and to ensure that these were included in the questionnaire. The topic guide was developed by the expert panel and asked open-ended questions phrased in a neutral manner such as, ‘How have you experienced the care at this clinic?’ and ‘What is your understanding of good or quality care for your chronic illness?’ The interviews were audio-recorded and transcribed verbatim and anonymised.

#### Analysis

Inductive descriptive analysis was carried out. Two researchers independently examined the transcripts and identified thematic categories. An iterative approach employing immersion and crystallisation was used to create a coding frame by each researcher. Coding allowed for breadth of experience and reported on both common and deviant views. Themes were checked for internal consistency. Once the researchers had independently created coding frames, the coding frames were compared for areas of interpretive disagreement to construct a consensus report including the complete coding frame with anonymised illustrative quotes to capture how patients construct a good experience of care.

### Phase 2b

The expert panel reviewed the results of Phases 1 and 2a and revised the consensus questionnaire of Phase 1 to incorporate patient needs. A subsequent questionnaire was developed (Table 1). The tool was translated into Afrikaans and isiXhosa by bilingual translators and used in Phase 2b. The purpose of the cognitive interviews in Phase 2b was to determine patient comprehension and interpretation of questions, as well as to determine whether patients found any questions confusing, upsetting or irrelevant and to confirm that all key areas were covered.

**TABLE 1:** A summary of the questions developed and in the order in which they were presented and used during the three phases of validation.

Phase 1 (expert consensus)	Phase 2b (incorporating themes from qualitative interviews)	Phase 3 (revised tool following cognitive interviews)
I have a good understanding of my chronic illness. I was given an opportunity to ask questions. When you go to your Primary Care Provider are you happy that you are taken care of by the same doctor or nurse each time? The concerns you raised were addressed to your satisfaction? Are your questions answered in ways that you understand? Does your Primary Care Provider give you enough time to talk about your worries or problems. Are you happy about that? In the past year, has the use of your medications been explained to you at the pharmacy? Do you feel comfortable telling your Primary Care Provider about your problems and worries? Did you receive any health talks whilst you were waiting at the club? Do you feel that staff care about your health. Are you satisfied with your overall care?	I am treated with respect. I feel that staff care about my health. The nurse and/or doctor listened to my problems and worries. The nurse and/or doctor checked up on what was really bothering me. The nurse and/or doctor explained to me what to do to help my illness. I understand how I can help to improve my health. I am satisfied that I get the medication that I need. I am able to see the same nurse and/or doctor if I choose to. The waiting time was reasonable. I am satisfied with my overall care. If not, please indicate the reason.	I am satisfied with my overall care. If not, please indicate the reason. I am treated with respect. I feel that staff care about my health. The nurse and/or doctor listened to my problems and worries. The nurse and/or doctor checked up on what was really bothering me. The nurse and/or doctor explained to me what to do to help my illness. I understand how I can help to improve my health. I am satisfied that I get the medication that I need. I am able to see the same nurse and/or doctor if I choose to. The waiting time was reasonable.

#### Sampling

Thirty cognitive interviews with users of the primary care services were conducted by trained fieldworkers in three languages (10 English, 10 Afrikaans and 10 isiXhosa). The same four sites used in Phase 2a were used for the cognitive interviews. The researchers based at the facilities purposefully selected participants to represent different chronic diseases (diabetes, hypertension, asthma, COPD and epilepsy), as well as different language groups (English, isiXhosa and Afrikaans).

#### Data collection

The cognitive interviews were performed by an experienced fieldworker who is fluent in all three languages. The fieldworker was trained in the use of the cognitive interview guide. The interviews were audio-recorded and transcribed verbatim and anonymised.

#### Data analysis

Two researchers analysed the transcripts for how the questions had been interpreted. Following the cognitive interviews, the expert panel reviewed the results of Phase 2b and revised the questionnaires in three languages for use in Phase 3.

### Phase 3: Reliability

The objectives of Phase 3 were to test the internal consistency of the tool, timeframe to complete as well as floor and ceiling effects.

#### Sampling

During Phase 3, patients with chronic conditions at one CHC were randomly sampled and invited to participate in the survey. This CHC had been selected as the patient population allowed for the testing of the tool in three languages. Participants were sampled from clients who attend chronic disease clubs for diabetes, hypertension, asthma, COPD and epilepsy by taking a random sample of the chronic care folders at the pharmacy. The number of folders was divided by the number of interviews for that day. Counting started at a random number and then taking every *n*th folder.

#### Data collection

Two hundred questionnaires (draft tool version 3) were administered by two experienced fieldworkers in three languages. This number has been shown by Streiner and Normand^24^ to be sufficient to test internal consistency of a scale that has about 10 items, with Cronbach’s alpha (coefficient alpha). Data from the questionnaires were captured using Epidata and exported onto an Microsoft Excel spreadsheet.

#### Analysis

Stata IC version 10.1 was used to analyse the data. The data were used to calculate Cronbach’s alpha to determine internal consistency (reliability).^25^

The acceptability of the data set was assessed by analysing the distribution of patient responses and determining whether there were any floor or ceiling effects, where more than 15% of the respondents did not achieve the lowest or highest positive score.^26^

A broad distribution (median, range and interquartile range) of responses throughout the range of responses implies content validity.^27^ The distribution of the responses is an indication of the sensitivity of a tool. The time taken to complete the questionnaire was assessed as this factor is important in a clinical setting where the audits are conducted and the time constraints under which clinical staff, who will be conducting the audit, operate.

### Ethical considerations

The study was approved by the University of Cape Town Faculty of Health Sciences Human Research Ethics Committee (Ref:201/2013) and the Western Cape Government, Department of Health (Ref: 2013 RP068). Participants were informed about the aims and objectives of the study and informed about their right to refuse participation, with no effect on access to care if they declined. Participants received a copy of the information. Written, informed consent was obtained from all participants. This protocol complied with the Helsinki Declaration of 2013^28^.

## Results

### Phase 1: Consensus validity

The expert panel reviewed existing tools^18,19,20,21,22^ for suitability in the South African context. For the purpose of the Integrated Chronic Disease Audit of which this tool would form a part, a short questionnaire that was easy to complete was needed. Some of the existing tools were too long or complex and contained questions that were not relevant to the South African setting but rather to a setting where a specific CCM had been implemented. The consensus questionnaire is presented in Table 1.

### Phase 2: Face validity

#### Phase 2a

**FIGURE 2:**
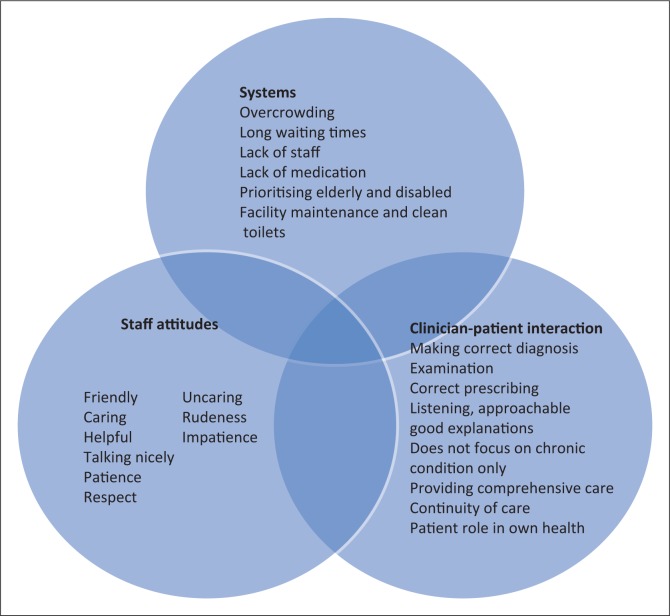
A summary of the three major themes emerging from semi-structured interviews exploring what patients considered to be quality chronic care.

The purposeful sample consisted of patients with hypertension (nine), diabetes (eight), asthma (four), HIV (one) and other chronic conditions (seven) such as gout, osteoarthritis, rheumatoid arthritis and ischaemic heart disease. Six of the 20 participants had two or more chronic conditions. The results of the thematic analysis of patients’ responses are summarised in Figure 2. Three major themes emerged from the investigation of what patients considered to be good quality chronic care, namely staff attitudes, clinician–patient interactions and systems at the facilities. Patients perceived the way in which they were treated by staff and clinicians to be more important than the technical aspects of the care that they received at the health centres.

Regarding staff attitudes, there were positive and negative responses. Positive comments included:

‘They’ve got patience, you know what I mean, they know how to listen.’ (B5)

‘… it doesn’t mean because you poor you must be treated in a different way.’ (D1)

‘The thing makes me feel welcome is this – ok is the way you talk with the nurse or the way the doctor talk to you, the most thing, respect you know.’ (B3)

Negative comments about staff attitudes included:

‘… they don’t care you see, they not busy, they sit and chat’ (B5)

‘I mean it’s not right we not animals, we’re people and we coming here for help, it’s not right what they doing to us.’ (D2)

‘Sometimes the sister in front is very rude there.’ (D4)

‘They don’t talk nicely the people, they shouting.’ (C2)

Patients had specific ideas about the clinician–patient interaction, expressing a wish to be examined:

‘… [*w*]e like to be touched, we like to be examined, like now it’s just now a conversation I would say, I feel sometimes if doctor was attending me and you just look on the chart and you write, and prescribe whatever you prescribe and all that without examining me, then I always think “Ay, something is not happy”.’ (C3)

‘Exam, you know not just sit on a chair and asking me what’s wrong and then its finish? Examine me, then I’ll be satisfied.’ (C4)

‘Systems at the facilities were commented on with waiting times standing out: “Waiting times”, “Not let me wait a long time because you know I’m full of pains sitting on the chair.”(C4)

‘To come out a little bit earlier, because we come early in the morning.’ (B4)

#### Phase 2b

The expert panel reviewed the results of Phases 1 and 2a and revised the consensus questionnaire of Phase 1 to incorporate patient needs. The questionnaire developed for the cognitive interviews in Phase 2b (Table 1) focused on staff attitudes (questions 1–2), the clinician–patient interaction (questions 3–4), patient empowerment (questions 5–6) and system issues (questions 7–9). The panel developed the questions in simple language that would be easy for patients to understand and it was translated into Afrikaans and isiXhosa.

Two of the 10 English interviews were not used as one was incomplete and a second was excluded because of poor recording quality. The transcripts of the 28 interviews analysed, indicated a good interpretation of questions. It was found that the order of questions influenced the responses and the order of questions was therefore changed in the final phase. Question 9 (waiting time) evokes strong responses that influenced the answers to question 10 (satisfaction with overall care). In the Phase 3 questionnaire, the question about satisfaction with overall care, with an open-ended section, was moved to the beginning of the questionnaire as question 1 and the question about waiting time became question 10. Patient responses to questions distinguished between clinicians, that is, doctors and clinical nurse practitioners (CNPs). It was decided not to alter the questions as CNPs were often the primary health providers at small CHCs where there were no resident doctors. The integrated Chronic Disease Audit of which the tool will form a part is conducted in all the primary care facilities in the Western Cape that provide chronic care, including small CHCs.

### Phase 3: Internal consistency and time taken to complete

Two hundred patients attending for chronic care at Vanguard CHC were surveyed in this phase, as the patient population at this CHC includes all three language groups studied. This group consisted of 48 men and 152 women. The ages of the participants ranged from 23 to 82 years, the median age was 56. The patients were surveyed in the three main mother tongue languages of the patients, namely Afrikaans (*n* = 61), English (*n* = 82) and isiXhosa (*n* = 57).

Internal consistency was determined using the Cronbach’s alpha coefficient, alpha values for the 10 items revealed acceptable reliability (the range is between 0.84 and 0.85), the overall Cronbach’s alpha was 0.86. This was considered to be good internal consistency.^25^ For the time taken to complete the survey, the mean was 3.8 ± 1.9 min with a range of 1–11 min and the median was 3 min.

**FIGURE 3:**
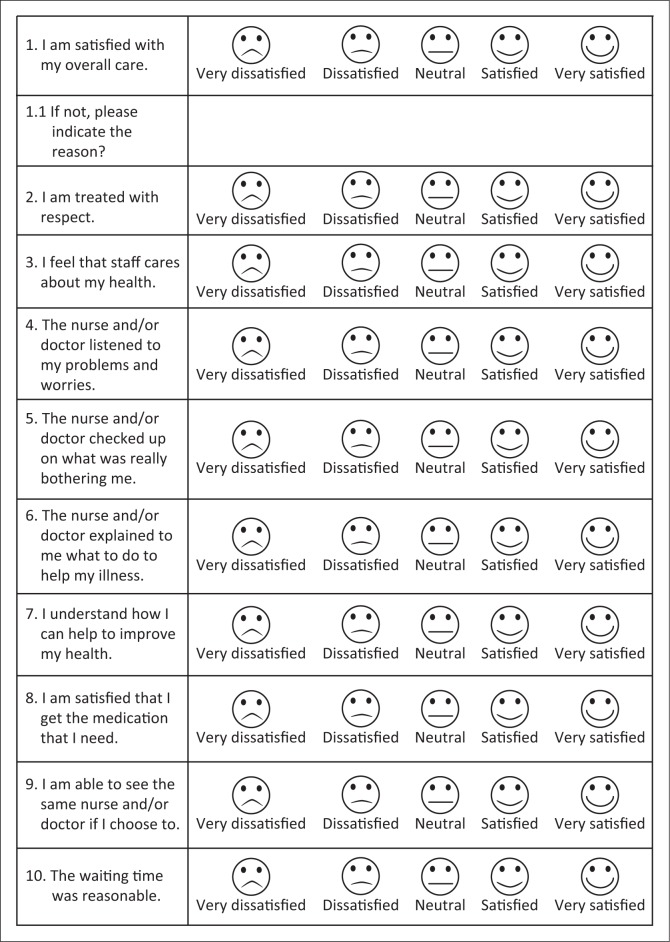
Final tool.

The distribution of patient responses were analysed for ceiling or floor effects. No floor effects were found (i.e. more than 15% of the respondents did not achieve the lowest positive score^26^). A ceiling effect was found for questions 4 (listened) and 8 (medication), with 20% of respondents giving a maximum score of 5 for question 4 and 17.5% for question 8. The distribution was skewed to the right indicating that patients are satisfied with their care (Table 2). One participant did not complete question 2, the other 199 participants completed all 10 questions. Following Phase 3, the questionnaire was finalised (see Figure 3).

**TABLE 2:** The distribution of patient responses (see bigger version attached in separate document).

Response option	Q1		Q2		Q3		Q4		Q5		Q6		Q7		Q8		Q9		Q10	
	*n*	%	*n*	%	*n*	%	*n*	%	*n*	%	*n*	%	*n*	%	*n*	%	*n*	%	*n*	%
Very dissatisfied	13	6.5	7	35	9	45	2	1	1	0.5	2	1	1	0.5	3	3	13	6.5	70	35
Dissatisfied	39	19.5	22	11	27	13.5	11	5.5	28	14	12	6	15	7.5	19	9.5	45	22.5	41	20.5
Neutral	24	12	12	6	17	8.5	16	8	16	8	14	7	9	4.5	5	5	19	9.5	5	2.5
Satisfied	99	49.5	130	65	124	62	131	65.5	125	62.5	150	75	146	73	139	69	109	54.5	72	36
Very satisfied	25	12.5	28	14	23	11.5	40	20	30	30	22	11	29	14.5	35	17.5	14	7	11	5.5
No response	0	-	1	0.5	0	-	0	-	0	-	0	-	0	-	0	-	0	-	0	-
**Total**	**200**	**100**	**200**	**100**	**200**	**100**	**200**	**100**	**200**	**100**	**200**	**100**	**200**	**100**	**200**	**100**	**200**	**100**	**200**	**100**

Q, question

## Discussion

This paper describes the process to develop a validated tool (PREM) to access patient experience of chronic care in the South African health care setting. Questions from existing questionnaires^18,19,20,21,22^ combined with themes from qualitative interviews with patients resulted in a tool that is locally relevant. The PCAT questionnaire has since been validated for South Africa.^29,30^ It is a comprehensive tool for assessing the primary health care performance in multiple domains, whereas the Chronic Care Assessment of Patient Experience (CCAPE) tool described in this study was designed as a short tool that was easy to administer.

This study found that a patient experience questionnaire in the patient’s mother tongue was a valid and reliable tool with good psychometric properties. Three major themes emerged from the investigation of what patients considered to be good quality chronic care, namely staff attitudes, clinician–patient interactions and systems at the facilities. Patients perceived the way in which they were treated by staff and clinicians to be more important than the technical aspects of the care that they received at the health centres. Gilson^31^ commented that:

‘Health systems are inherently relational and so many of the most critical challenges for health systems are relationship problems. Poor staff attitudes towards patients can cause dissatisfaction with services, which even good technical care may not offset.’

Patients had specific ideas about the clinician–patient interaction, expressing a wish to be examined. Systems at the facilities were commented on with waiting times being the most problematic. This is similar to the findings of a population-based survey of health system responsiveness in South Africa that found ‘waiting time for care’ had the lowest score.^32^

The focus on staff attitudes, the clinician–patient interaction, patient empowerment and system issues in the questionnaire developed for Phase 2b (Table 1) is aligned with requirements for quality care for chronic diseases.^33,34^ Patient empowerment and self-management has been recognised as an important aspect of chronic disease care.^35^

This tool has been tested for comprehension in the Phase 2b in the three main mother tongue languages of the patients, as well as for aspects of reliability in Phase 3. Cronbach’s alpha coefficient was used to determine internal consistency, with the overall Cronbach’s alpha 0.86. This was considered to be good internal consistency.^25^ The median time taken to complete the survey of 3 min is satisfactory given the original intention of the researchers that the questionnaire should be quick and easy to complete as part of an annual audit without placing huge strain on the staff at the primary care facilities.

The combination of questions from existing questionnaires, the rigorous process followed in drawing up the final questionnaire, combined with themes from qualitative interviews with patients resulted in a questionnaire that is locally relevant. The questions were easy to understand and the pictorial response made it easy for patients to choose the most appropriate response. The new tool has been incorporated into the annual Chronic Disease Audit for the Western Cape and gives a voice to the patients’ perceptions about the health services delivered to them. The responses obtained, guide the health authorities on how to improve the delivery of health care to this population. Patients’ evaluation of care has been shown to be a realistic tool to provide opportunity for improvement, enhance strategic decision-making, reduce cost, meet patients’ expectations, frame strategies for effective management, monitor health care performance of health plans and provide benchmarking across health care institutions.^36^ A recent publication describes a tool to measure patient experience of chronic care that was developed in Spain.^37^ The authors of this Spanish study conclude that measurement of the patient experience of chronic illness care can facilitate health systems’ reorientation towards integrated patient-centred care.^37^

A limitation of this study is that the tool has only been used in the Western Cape. The interviewers for Phase 2a were on the expert panel, which may have influenced the results when combining Phases 1 and 2a. Construct validity, test–retest reliability and responsiveness were not tested in this study. Future studies will enable that to be tested.

## Conclusion

Improvement of the quality of chronic care delivery should always be accompanied by investment in the quality of relationships and communication between patients and professionals.^34^ This PREM is one way to measure patient’s experiences of this relationship. The authors have named it CCAPE. It can become a tool which can be applied across South Africa and beyond, if it is translated into local languages. Implementation studies are now required to determine how best to use this in routine practice, and further research can focus on the impact of using PREMs in the improvement of chronic care.
